# FAS and FASL expression in autoimmune lymphoproliferative syndrome and immune thrombocytopenia: limited diagnostic utility as transcriptional biomarkers

**DOI:** 10.1007/s11033-026-12950-0

**Published:** 2026-10-16

**Authors:** Paola Montanari, Federico Rondot, Anna Pau, Anna Clemente, Cristina Calvi, Ilaria Galliano, Stefano Gambarino, Massimiliano Bergallo

**Affiliations:** 1 Department of Public Health and Pediatric Sciences, Medical School, Immunopathology Laboratory, University of Turin, Turin, 10126 Italy; 2 Laboratory of Specialistic Pediatry, Department of Children’s Disease and Care, Regina Margherita Children Hospital, Turin, 10126 Italy; 3 Department of Molecular Biotechnology and Health Sciences, University of Turin, Turin, 10126 Italy

**Keywords:** Autoimmune lymphoproliferative syndrome, Immune thrombocytopenia, FAS, FAS ligand, Apoptosis, Biomarkers

## Abstract

**Background:**

Autoimmune lymphoproliferative syndrome (ALPS) is an immune dysregulation disorder caused by defective lymphocyte apoptosis, most commonly involving the FAS–FAS ligand pathway. Because ALPS frequently presents with autoimmune cytopenias, particularly immune thrombocytopenia (ITP), its diagnosis may be challenging. This study evaluated whether FAS and FAS ligand gene expression could distinguish ALPS from ITP.

**Methods:**

FAS and FAS ligand messenger RNA expression was measured by quantitative real-time polymerase chain reaction in whole-blood samples from patients with pediatric-onset ALPS (*n* = 24), patients with chronic ITP (*n* = 27), and healthy controls (*n* = 22). Expression levels were compared among groups, and their diagnostic performance was assessed using receiver operating characteristic curve analysis.

**Results:**

FAS expression was significantly higher in patients with ALPS than in healthy controls (*p* < 0.01), but no significant difference was observed between ALPS and ITP. FAS expression showed moderate discrimination between ALPS and healthy controls but poor discrimination between ALPS and ITP. FAS ligand expression did not differ significantly among groups and showed no meaningful diagnostic performance. Neither FAS nor FAS ligand expression correlated with double-negative T-cell percentages or differed according to treatment status in patients with ALPS.

**Conclusions:**

FAS and FAS ligand transcriptional levels do not provide disease-specific discrimination between ALPS and ITP. These findings support the continued use of clinical, immunophenotypic, functional, and genetic investigations for the diagnosis of ALPS.

**Supplementary Information:**

The online version contains supplementary material available at https://doi.org/10.1007/s11033-026-12950-0.

## Introduction

Autoimmune Lymphoproliferative Syndrome (ALPS) represents a prototypical form of immune dysregulation, characterized by impaired lymphocyte apoptosis, chronic non-malignant lymphoproliferation, autoimmune manifestations, particularly autoimmune cytopenias, and an increased lifetime risk of lymphoma [1–3]. From a clinical standpoint, ALPS poses a significant diagnostic challenge, as its presentation frequently overlaps with more prevalent immune-mediated conditions, including immune thrombocytopenia (ITP), autoimmune hemolytic anemia, and Evans syndrome [2, 4, 5].

The classification of disorders presenting with lymphoproliferation and autoimmunity has recently been reconsidered. Magerus et al. proposed the term autoimmune lymphoproliferative immunodeficiencies (ALPID) to encompass a broader spectrum of immune dysregulatory conditions sharing these manifestations, including cases without an established molecular diagnosis. Within this framework, a revised definition of ALPS emphasizes demonstrable disruption of FAS signaling associated with characteristic clinical and biological findings. This distinction highlights the importance of integrating clinical, immunophenotypic, functional, and genetic information to differentiate ALPS from other disorders with overlapping presentations [6].

At the molecular level, ALPS is primarily linked to abnormalities in the FAS-mediated apoptotic pathway. The FAS gene (TNFRSF6, also known as CD95) encodes a cell-surface death receptor that plays a key role in activation-induced cell death, a fundamental mechanism for maintaining peripheral immune tolerance [7–9]. Germline or somatic mutations in FAS account for most genetically defined ALPS cases [1, 10]. These alterations impair the elimination of autoreactive or excess lymphocytes, thereby promoting lymphoproliferation and autoimmunity [7, 8]. CASP10 variants were historically included in the genetic classification of ALPS [1, 11]; however, their causal role has been challenged by recent functional evidence. Consonni et al. demonstrated that the investigated missense variants, as well as a homozygous frameshift variant abolishing CASPASE-10 protein expression, did not impair FAS-mediated apoptosis [12].

Despite this well-established pathogenic framework, the translation of molecular insights into practical diagnostic tools remains limited. Current diagnostic algorithms for ALPS rely on a combination of clinical features, immunophenotyping, particularly the expansion of TCRαβ⁺ CD4⁻CD8⁻ double-negative T cells, functional apoptosis assays, and genetic testing [1, 6, 13–16]. However, many of these investigations are not readily available in routine clinical practice, particularly outside specialized referral centers. Consequently, ALPS may remain underdiagnosed or be misclassified as isolated immune cytopenia, especially at disease onset [2, 4, 5].

In this context, transcriptional analysis of genes involved in the apoptotic pathway, including FAS and FASL, may represent a potential diagnostic adjunct. This hypothesis is supported by the central pathogenic role of the FAS–FASL axis in ALPS [6–10]. Nevertheless, immune-cell activation can influence FAS ligand expression, membrane trafficking, and release, raising concerns regarding the specificity of gene expression-based biomarkers [17–19].

Clarifying whether FAS and FASL expression profiles can distinguish ALPS from clinically overlapping conditions such as ITP is therefore of direct clinical relevance. The present study compared FAS and FASL expression levels in patients with ALPS, patients with chronic ITP, and healthy controls to evaluate their potential diagnostic and translational value.

## Materials and methods

### Patient selection

Peripheral blood samples collected in ethylenediaminetetraacetic acid tubes were obtained from three study groups: patients fulfilling diagnostic criteria for autoimmune lymphoproliferative syndrome (ALPS), patients with chronic immune thrombocytopenia (ITP), and healthy controls (HC). All patients with ALPS had disease onset during childhood; however, some had reached adulthood at the time of blood sampling.

Patients were classified as having ALPS based on the presence of persistent, non-malignant, non-infectious lymphoproliferation, documented by lymphadenopathy and/or splenomegaly lasting for at least 6 months, together with an increased proportion of circulating TCRαβ⁺ CD4⁻CD8⁻ double-negative T cells exceeding 2.5% of CD3⁺ T lymphocytes. Twenty-four patients met these criteria and were included in the ALPS group. At the time of blood collection, seven patients were receiving immunomodulatory treatment with mycophenolate mofetil or sirolimus for the control of lymphoproliferation and associated cytopenias.

The ITP group included 27 pediatric patients with thrombocytopenia persisting for more than 12 months. None showed clinical signs of lymphoproliferation or laboratory findings suggestive of ALPS, including increased double-negative T-cell percentages.

The HC group included 22 age-comparable, asymptomatic individuals undergoing routine laboratory testing at Regina Margherita Children’s Hospital, Turin, Italy, all of whom had results within the established reference ranges. Individuals were excluded in the presence of known or suspected infectious, malignant, autoimmune, inflammatory, neurological, or other pathological conditions, or abnormal laboratory findings.

Clinical and demographic characteristics of the study population are summarized in Table 1.

**Table 1:** Clinical and demographic characteristics of the study population

Variable	HC (*n* = 22)	ALPS (*n* = 24)	ITP (*n* = 27)
Age (years): median (IQR)	11.7 (8.4–17.0)	15.9 (13.2–28.1)	12.4 (8.9–15.3)
Male sex: *n* (%)	14 (63.6)	15 (62.5)	13 (48.1)
Platelet count (×10⁹/L): median (IQR)	256 (190–418)	76 (54–139)	51 (25–115)
DNT cells (% CD3+): median (IQR)	NA	4.2 (3.1–6.8)	0.9 (0.5–1.6)
Lymphadenopathy and/or splenomegaly: *n* (%)	NA	24 (100)	0 (0)
Ongoing treatment: *n* (%)	NA	7 (29.2)	0 (0)
Disease duration (years): median (IQR)	NA	8.0 (3.5–16.2)	3.3 (1.6–6.8)

HC = Healthy Controls; ALPS = Autoimmune lymphoproliferative syndrome; ITP = immune thrombocytopenia; DNT cells = CD4⁻CD8⁻ double-negative T cells; NA = not applicable; n = number; % = percentage; IQR = interquartile range

The three groups were comparable in terms of age (Kruskal–Wallis test, p = 0.8622) and sex distribution (chi-square test, p = 0.4605)

All analyses were performed using residual material from routine clinical samples after written informed consent had been obtained from adult participants or from parents or legal guardians of participants younger than 18 years. Data were anonymized before analysis. The study was conducted in accordance with the Declaration of Helsinki and was approved by the Ethics Committee of Azienda Ospedaliera Universitaria Città della Salute e della Scienza di Torino (approval no. 00313, 23 July 2020).

### Reverse transcription

Total RNA was isolated from whole-blood samples using the Maxwell^®^ automated extraction system and the Maxwell^®^ RNA Blood Kit (Promega, Madison, WI, USA), according to the manufacturer’s instructions. DNase treatment was included to remove contaminating genomic DNA. RNA concentration and purity were assessed using a NanoDrop™ 2000 spectrophotometer (Thermo Fisher Scientific, Waltham, MA, USA).

For complementary DNA synthesis, 500 ng of total RNA was reverse-transcribed in a final volume of 20 µL using ImProm-II™ Reverse Transcriptase (Promega, Madison, WI, USA), random hexamer primers, and the supplied reaction components. Reverse transcription was performed on a GeneAmp^®^ PCR System 9700 under the following conditions: 5 min at 25 °C, 60 min at 42 °C, and 15 min at 70 °C. Complementary DNA samples were stored at − 20 °C until analysis.

No-reverse-transcriptase controls were included to verify the absence of genomic DNA contamination.

### Real time PCR

FAS and FASL messenger RNA expression levels were quantified by probe-based quantitative real-time PCR. Glyceraldehyde-3-phosphate dehydrogenase (GAPDH) was used as the endogenous reference gene. Amplification and fluorescence detection were performed using an ABI PRISM^®^ 7500 Real-Time PCR System (Life Technologies, Carlsbad, CA, USA).

Each reaction contained 50 ng of complementary DNA in a final volume of 20 µL, including GoTaq^®^ Probe qPCR Master Mix (Promega, Madison, WI, USA), primers at a final concentration of 500 nM, and probes at 200 nM. Primer and probe sequences for GAPDH, FAS, and FASL are reported in Table 2.

**Table 2:** Primer and Probe sequences used in this study

Target	Primers	Probes
GAPDH	PF 5’-CGAGATCCCTCCAAAATCAA-3’ PR 5’- TTCACACCCATGACGAACAT-3’	6FAM-TGGAGAAGGCTGGGGCTCAT-TAMRA
FAS	PF 5’- TGAAGGACATGGCTTAGAAGTG − 3’ PR 5’- GGTGCAAGGGTCACAGTGTT − 3’	6FAM-AAACTGCACCCGGACCCAGAATACC-TAMRA
FASL	PF 5’- GCAGCCCTTCAATTACCCAT − 3’ PR 5’- CAGAGGTTGGACAGGGAAGAA − 3’	6FAM-TCCCCAGATCTACTGGGTGGACAGC-TAMRA

Primers and probes were designed using Primer Express™ software, version 3.0 (Applied Biosystems, Foster City, CA, USA), and their specificity was verified in silico using the Basic Local Alignment Search Tool.

Thermal cycling conditions consisted of an initial denaturation step at 95 °C for 2 min, followed by 45 cycles at 95 °C for 15 s and 60 °C for 1 min. No-reverse-transcriptase controls were included to exclude genomic DNA contamination.

Relative gene expression was calculated using the comparative threshold cycle method and expressed as relative quantification values.

### Statistical analysis

Differences in FAS and FASL expression levels among the three study groups were assessed using the Kruskal–Wallis test. When the overall test was statistically significant, Dunn’s multiple-comparisons test was used for post hoc pairwise comparisons. Continuous clinical and demographic variables were compared using the Kruskal–Wallis test, whereas categorical variables were analyzed using the chi-square test or Fisher’s exact test, as appropriate.

Correlations between gene expression levels and clinical parameters were assessed using Spearman’s rank correlation coefficient. FAS and FASL expression levels in treated and untreated patients with autoimmune lymphoproliferative syndrome were compared using the Mann–Whitney U test.

Receiver operating characteristic curves were generated to evaluate the ability of FAS and FASL expression levels to discriminate patients with autoimmune lymphoproliferative syndrome from patients with immune thrombocytopenia and from healthy controls. Diagnostic performance was expressed as the area under the curve with the corresponding 95% confidence interval.

All tests were two-sided, and a *p* value < 0.05 was considered statistically significant. Analyses were performed using GraphPad Prism version 7.0 (GraphPad Software, La Jolla, CA, USA).

## Results

### FAS gene expression

**Fig. 1:**
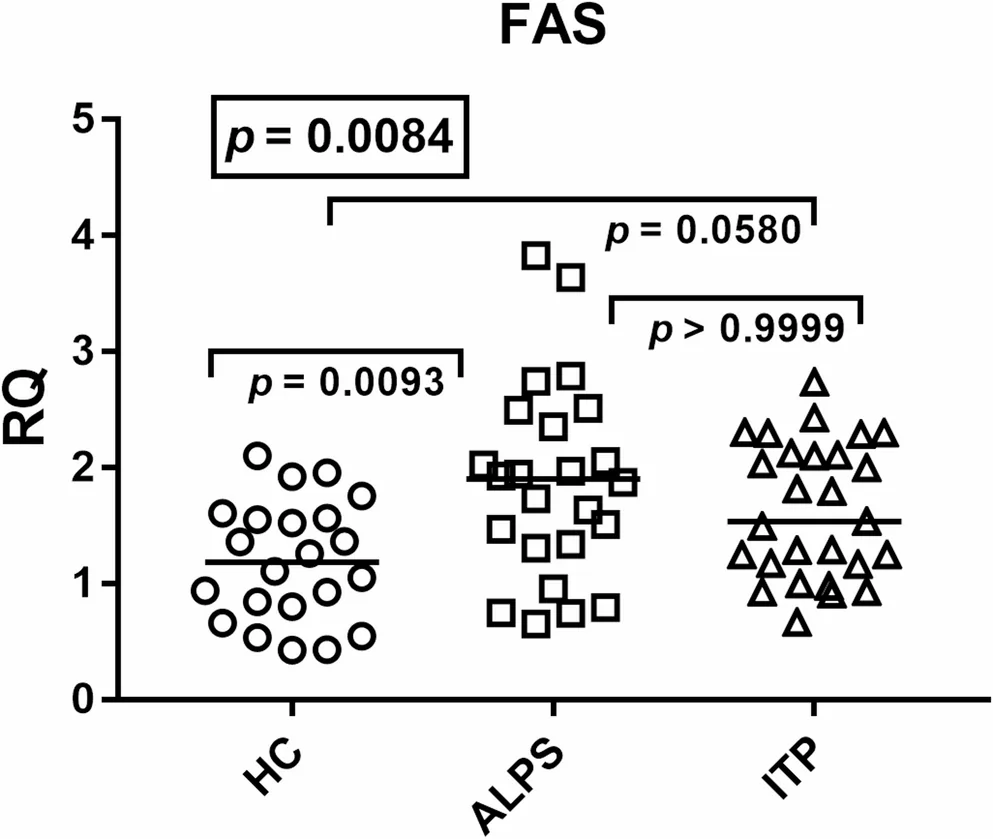
Whole-blood FAS mRNA expression in healthy controls (HC), patients with autoimmune lymphoproliferative syndrome (ALPS), and patients with immune thrombocytopenia (ITP). Expression is reported as relative quantification (RQ) values normalized to GAPDH. Each symbol represents one participant; horizontal lines indicate medians. The boxed p value refers to the overall Kruskal–Wallis test. Brackets indicate pairwise comparisons assessed using Dunn’s multiple-comparisons test, with multiplicity-adjusted *p* values

The transcriptional expression of FAS was significantly different among the three study groups (Kruskal–Wallis test, *p* = 0.0084) (Fig. 1).

Patients with ALPS exhibited significantly higher FAS mRNA expression levels compared with healthy controls (HC) (*p* = 0.0093).

No statistically significant difference was observed between ALPS patients and subjects with immune thrombocytopenia (ITP) (*p* > 0.9999).

FAS expression in ITP showed a non-significant trend toward higher values compared with the HC groups (*p* = 0.0580).

Overall, these findings suggest that FAS transcription is increased in ALPS compared with healthy controls but does not distinguish ALPS from ITP, with substantial overlap in expression levels between the two groups.

Median and IQR (25%–75%) values were 1.18 (0.81–1.56) for HC, 1.93 (1.32–2.42) for ALPS, and 1.53 (1.17–2.11) for ITP.

### FASL gene expression

**Fig. 2:**
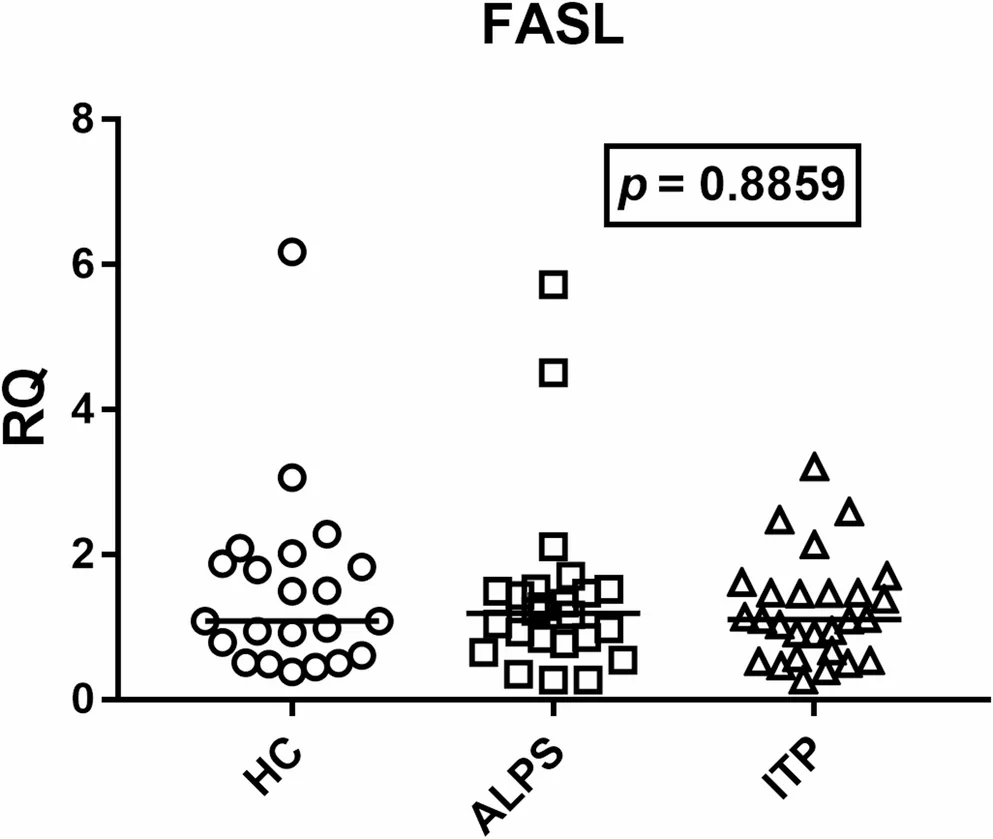
Whole-blood FASL mRNA expression in healthy controls (HC), patients with autoimmune lymphoproliferative syndrome (ALPS), and patients with immune thrombocytopenia (ITP). Expression is reported as relative quantification (RQ) values normalized to GAPDH. Each symbol represents one participant; horizontal lines indicate medians. The boxed *p* value refers to the overall Kruskal–Wallis test

In contrast to FAS, FASL mRNA expression levels were comparable among ALPS patients, ITP subjects, and healthy controls, with no statistically significant differences observed across groups (Kruskal–Wallis test, *p* = 0.8859) (Fig. 2).

Median and IQR (25%–75%) values were 1.08 (0.66–1.87) for HC, 1.22 (0.85–1.51) for ALPS, and 1.10 (0.62–1.47) for ITP.

### Correlation analysis

To further explore the potential clinical relevance of transcriptional findings, correlation analyses were performed between FAS/FASL expression levels and DNT cell percentage in ALPS patients.

**Fig. 3:**
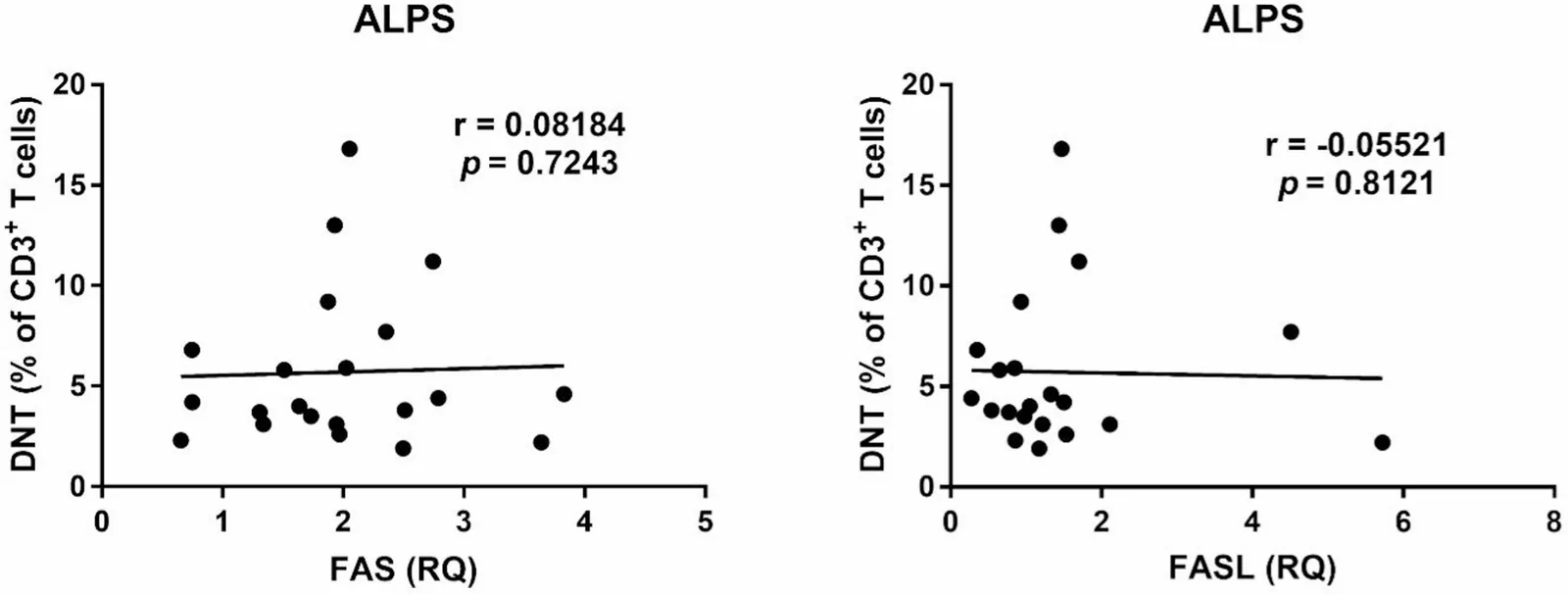
Correlation between whole-blood FAS (left panel) or FASL (right panel) mRNA expression and double-negative T-cell (DNT) percentages in patients with autoimmune lymphoproliferative syndrome (ALPS). Expression is reported as relative quantification (RQ) values normalized to GAPDH. Double-negative T-cells are defined as TCRαβ⁺ CD4⁻CD8⁻ cells and expressed as a percentage of CD3⁺ T cells. Each point represents one participant. Spearman’s correlation coefficients and two-sided p values are shown. Solid lines represent linear regression fits displayed as visual guides

No significant correlation was observed between FAS expression and DNT cell percentage (Spearman’s *r* = 0.08, *p* = 0.7243) (Fig. 3). Similarly, FASL expression was not associated with DNT cell percentage (*r* = − 0.06, *p* = 0.8121) (Fig. 3).

In addition, no significant differences in FAS or FASL expression were observed between treated and untreated ALPS patients (Mann–Whitney test, *p* = 0.4475 and *p* = 0.8907, respectively) (Supplementary Figure S1).

### ROC curve analysis

Receiver operating characteristic (ROC) curve analysis was performed to evaluate the diagnostic performance of FAS and FASL expression levels.

FAS expression showed moderate discriminative ability between ALPS patients and healthy controls, with an area under the curve (AUC) of 0.742 (95% CI: 0.600–0.885; *p* = 0.0049).

**Fig. 4:**
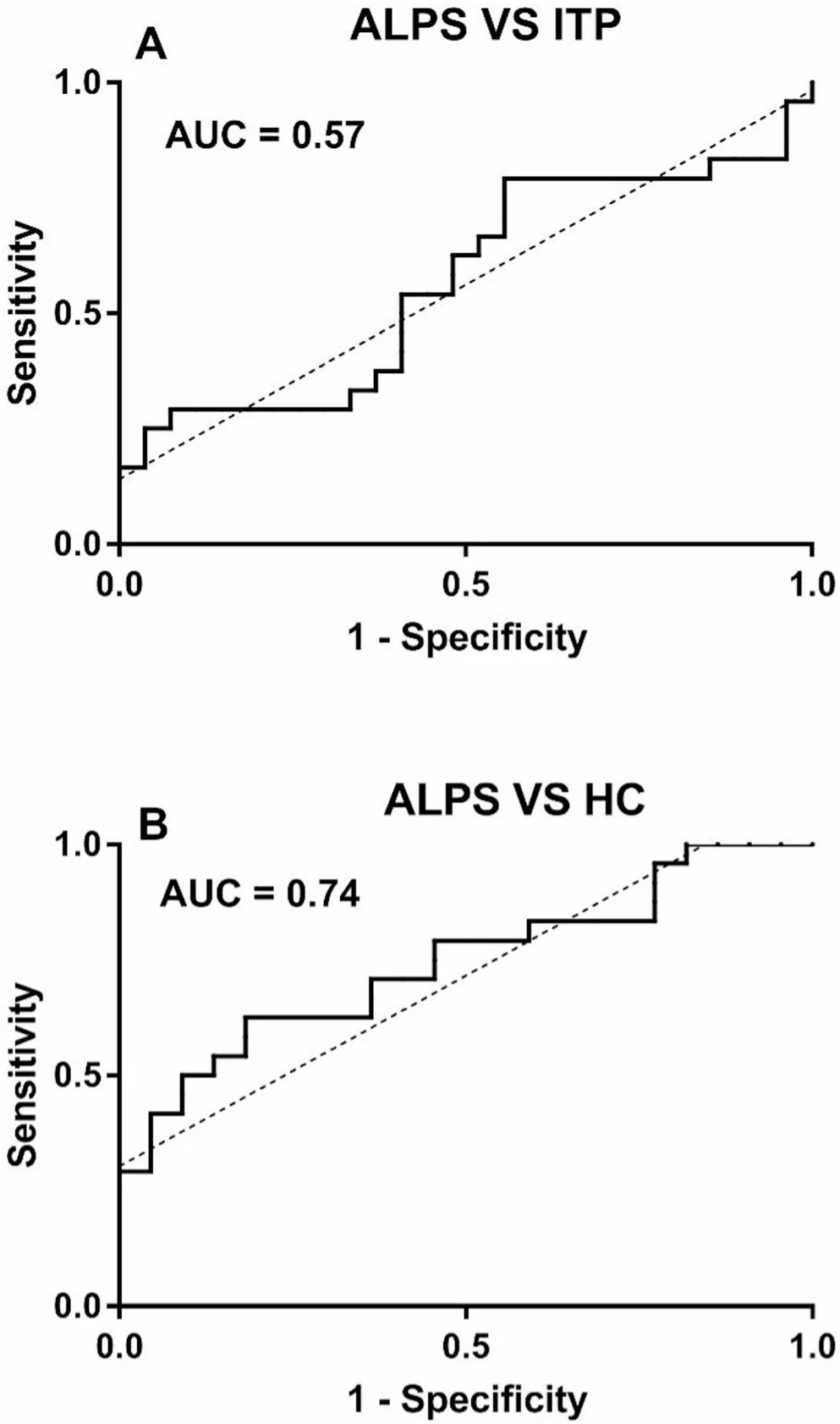
Receiver operating characteristic (ROC) curves for whole-blood FAS mRNA expression in distinguishing patients with autoimmune lymphoproliferative syndrome (ALPS) from (**A**) patients with immune thrombocytopenia (ITP) and (**B**) healthy controls (HC). Areas under the curve (AUCs) are shown within each panel. Dashed diagonal lines indicate no discrimination (AUC = 0.5)

In contrast, FAS expression demonstrated poor performance in distinguishing ALPS from ITP patients (AUC = 0.568; 95% CI: 0.405–0.731; *p* = 0.406), indicating limited diagnostic utility in a clinically relevant setting (Fig. 4).

FASL expression did not demonstrate meaningful discriminative ability in either comparison, showing no significant performance in distinguishing ALPS from ITP patients (AUC = 0.517; 95% CI: 0.356–0.678; *p* = 0.836) or from healthy controls (AUC = 0.534; 95% CI: 0.362–0.706; *p* = 0.692).

## Discussion

From a clinical–diagnostic standpoint, one of the principal challenges in the evaluation of patients with suspected Autoimmune Lymphoproliferative Syndrome (ALPS) lies in distinguishing this rare immune dysregulation disorder from more prevalent immune-mediated cytopenias. Autoimmune thrombocytopenia is particularly problematic in this regard, as it may occur either as an isolated condition or represent an early, and sometimes sole, manifestation of ALPS, leading to diagnostic uncertainty and delayed recognition of the syndrome [1–5]. In this context, the identification of accessible molecular markers capable of supporting early diagnostic discrimination would be of substantial clinical utility.

Although ALPS is mechanistically linked to defects in the FAS–FASL apoptotic pathway, our results indicate that transcriptional analysis of these genes has limited discriminatory value in this clinical setting. FAS expression was significantly increased in patients with ALPS compared with healthy controls; however, no statistically significant difference was observed between ALPS and immune thrombocytopenia (ITP), with substantial overlap in expression values. FAS expression in ITP showed only a non-significant trend toward higher levels compared with healthy controls. Receiver operating characteristic analysis confirmed these findings, showing moderate discrimination between ALPS and healthy controls but poor discrimination between ALPS and ITP. FASL expression did not differ significantly among groups and showed no meaningful diagnostic performance. Moreover, neither FAS nor FASL expression correlated with double-negative T-cell percentages or differed according to treatment status.

From a methodological perspective, the present study was intentionally designed to evaluate transcriptional readouts in whole blood, as these represent a minimally invasive and clinically accessible approach. Although functional and protein-level assays may provide deeper mechanistic insights, their limited availability in routine clinical practice, particularly outside specialized centers, can restrict their use as diagnostic tools [1, 2, 6, 13–16]. This highlights the relevance of evaluating simpler biomarkers while recognizing their inherent limitations. Our findings indicate that whole-blood transcriptional analysis of FAS and FASL alone is insufficient to discriminate ALPS from ITP in this clinical setting.

These findings highlight a key distinction: transcript abundance does not necessarily reflect protein function or apoptotic pathway activity. In ALPS, pathogenic FAS variants can impair receptor function and downstream signaling without necessarily reducing transcript levels [7, 8, 10, 20]. Preserved or increased FAS messenger RNA levels may therefore coexist with markedly impaired apoptotic responses. Compensatory transcriptional responses or chronic lymphocyte activation could contribute to the observed increase, although these mechanisms were not investigated in this study. The recent reassessment of CASP10 further illustrates the need for functional evidence when interpreting alterations in apoptosis-related genes: Consonni et al. found that the investigated CASP10 variants, including a variant abolishing CASPASE-10 protein expression, did not impair FAS-mediated apoptosis [12]. These findings challenge the historical attribution of a causal role to CASP10 variants in ALPS.

Our previous study investigated whole-blood CASP8 and CASP3 expression in patients with clinically diagnosed ALPS, patients with chronic ITP, and healthy controls [21]. Both transcripts were increased in the patient groups compared with controls; CASP8 expression was lower in ALPS than in ITP, whereas CASP3 expression did not differ significantly between the two diseases. The present study evaluates FAS and FASL transcription and found no significant differences between ALPS and ITP for either gene. These observations indicate that transcriptional differences between the two conditions vary across components of the apoptotic pathway. However, the CASP8 difference reported previously does not by itself establish diagnostic utility, and neither study directly links transcript abundance to protein activation or functional apoptotic responses.

This distinction is also relevant to other inborn errors of immunity, in which qualitative signaling defects cannot necessarily be inferred from messenger RNA expression alone [22, 23]. In these conditions, reliance on transcriptional biomarkers may oversimplify disease mechanisms and fail to capture functionally relevant abnormalities that are essential for accurate diagnosis.

The proposed ALPID framework further emphasizes the distinction between a shared clinical phenotype and a specific underlying disease mechanism [6]. In this framework, ALPS is distinguished from other autoimmune lymphoproliferative conditions by evidence of disrupted FAS signaling together with characteristic clinical and biological findings. The lack of discrimination between ALPS and ITP by FAS and FASL transcript measurements in our cohort illustrates the limitations of these readouts for differential diagnosis. Transcriptional analysis cannot establish functional impairment of FAS signaling or determine whether patients meet the proposed revised definition of ALPS.

FASL expression did not differ significantly among patients with ALPS, patients with ITP, and healthy controls. FAS ligand activity is regulated at post-transcriptional and post-translational levels, including membrane trafficking, proteolytic cleavage into soluble forms, and spatial regulation at the immune synapse [17–19, 24]. These mechanisms are not captured by bulk messenger RNA measurements and provide a biological rationale for distinguishing FASL transcript abundance from ligand activity.

Several methodological limitations should be acknowledged. First, the relatively small sample size, particularly in the ALPS group, reflects the rarity of the disease and the single-center design [1–4]. This may have limited the statistical power to detect moderate differences between ALPS and ITP; therefore, the findings should be considered exploratory and require confirmation in larger, multicenter cohorts. Second, the present study focused on transcriptional measurements and did not include an integrated analysis of functional and genetic findings. This limits the interpretation of transcript abundance in relation to underlying defects in FAS signaling. Future studies integrating transcriptional, functional, and genetic data may provide a more comprehensive assessment. Third, gene expression was measured in whole blood, which contains a heterogeneous mixture of cell populations; consequently, cell-specific transcriptional changes, particularly within activated T-cell subsets involved in ALPS pathogenesis, may have been masked. Finally, seven patients with ALPS were receiving immunomodulatory treatment at the time of sampling, which may have influenced gene expression. However, FAS and FASL expression did not differ significantly between treated and untreated patients, although the small subgroup sizes limit definitive conclusions.

Taken together, these findings have clear implications for clinical practice. In patients presenting with autoimmune cytopenias, FAS and FASL expression should not be used as standalone biomarkers to support or exclude a diagnosis of ALPS. If considered, transcriptional data should be interpreted only within a comprehensive diagnostic framework that prioritizes clinical features, immunophenotypic markers such as double-negative T-cell expansion, functional apoptosis assays, and targeted genetic testing [1, 2, 6, 13–16].

From a translational perspective, the present study underscores the distinction between pathogenic relevance and biomarker utility. Although disruption of the FAS–FASL pathway is central to ALPS pathogenesis, this defect is primarily functional and cannot be reliably inferred from transcript abundance alone [6–10, 20]. Diagnostic strategies should therefore prioritize integrated approaches combining clinical, immunophenotypic, functional, and genetic data rather than relying on isolated transcriptional measurements [1, 2, 6, 13–16, 22, 23].

In conclusion, FAS and FASL expression levels did not provide disease-specific discrimination between ALPS and ITP in this cohort. These findings indicate that whole-blood transcriptional analysis of the FAS–FASL pathway has limited diagnostic utility and reinforce the central role of clinical assessment, immunophenotyping, functional apoptosis assays, and genetic testing in the diagnostic workup of ALPS [1, 2, 6, 13–16].

## Supplementary Information

Below is the link to the electronic supplementary material.

Supplementary Material 2

## Data Availability

The data presented in this study are available on request from the corresponding author (the data are not publicly available due to privacy restrictions).
