# Supplementary material for: FAS and FASL expression in autoimmune lymphoproliferative syndrome and immune thrombocytopenia: limited diagnostic utility as transcriptional biomarkers
Source: Mol Biol Rep. 2026 Oct 16;53(1):1730. doi: 10.1007/s11033-026-12950-0 (PMC13675357; doi:10.1007/s11033-026-12950-0)
Supplement: Supplementary file 1 [file 11033_2026_12950_MOESM2_ESM.docx]

**Supplementary Materials**

**Figure S1. Whole-blood FAS (left panel) and FASL (right panel) mRNA expression in patients with autoimmune lymphoproliferative syndrome (ALPS), grouped by the presence or absence of ongoing immunomodulatory treatment at blood sampling.** Expression is reported as relative quantification (RQ) values normalized to GAPDH. Each symbol represents one participant; horizontal lines indicate medians. Groups were compared using the two-sided Mann–Whitney U test.
